# Soy Sauce Odor Improves Upper Limb Motor Performance with Preliminary Evidence of Increased Alpha-Band Intermuscular Coherence Between Postural Muscles: An Exploratory Within-Subjects Crossover Study

**DOI:** 10.3390/brainsci16070737

**Published:** 2026-07-12

**Authors:** Yutaka Yano, Junichi Inatomi, Hajime Yamakage, Tsuyoshi Miyata, Yoshihiro Murata, Mutsuo Taniguchi, Fumino Okutani, Hideto Kaba, Masahiro Yamaguchi

**Affiliations:** 1 Faculty of Health Sciences, University of Kochi Health Sciences, Kochi 781-5103, Japan; yano@ko-ken-k3.ac.jp (Y.Y.);; 2 Department of Physiology, Kochi Medical School, Kochi University, Kochi 783-8505, Japan; 3 Clinical Research Center, National Hospital Organization Kyoto Medical Center, Kyoto 612-8555, Japan; 4 Department of Occupational Health, Kochi Medical School, Kochi University, Kochi 783-8505, Japan

**Keywords:** olfaction, upper limb motor performance, postural muscle coordination, electromyography (EMG), intermuscular coherence (IMC), sensorimotor integration

## Abstract

**Highlights:**

**What are the main findings?**

- Soy sauce odor improved postural motor performance by increasing reach distance in the modified functional reach test.
- In this exploratory study, soy sauce odor showed increased alpha-band intermuscular coherence between the serratus anterior and lumbar multifidus muscles during reaching.

**What are the implications of the main findings?**

- Soy sauce odor improved reaching performance in the mFRT, accompanied by an exploratory, within-condition increase in α-band intermuscular coherence between postural muscles. Because no significant interaction effect was found, this coherence change cannot be regarded as the cause of the behavioral improvement, and the interpretation requires confirmation in larger studies.
- These hypothesis-generating findings warrant replication in larger, adequately powered studies before any application of odor-based approaches to postural stability or motor control in clinical populations can be considered.

**Abstract:**

**Background/Objectives**: While odor stimuli can modify motor function, the underlying mechanisms remain unclear. This study examined the impact of olfactory stimuli on muscle activities involved in upper limb motor performance and balance in nine healthy young Japanese males. **Methods**: In this exploratory, within-subject crossover study, odors of soy sauce, β-phenylethyl alcohol (PEA, a component of rose odor), and water were used to assess their effects on motor function through the modified functional reach test (mFRT), which measures sitting balance during upper limb extension. Surface electromyography from four muscles (upper trapezius, anterior deltoid, serratus anterior, and lumbar multifidus) was recorded to analyze intermuscular coherence (IMC) before and after odor exposure. **Results**: Only soy sauce odor increased mFRT reach distance. Within-condition analysis revealed that soy sauce odor was associated with increased α-band IMC in the serratus anterior–lumbar multifidus pair at the overall reach and second half of reach (both Benjamini–Hochberg-corrected *p* < 0.05; Cohen’s d > 1.0), while no significant between-condition difference (Group × Time interaction) was observed. No significant within-condition changes were observed for PEA or water. **Conclusions**: These preliminary findings suggest that soy sauce odor improved mFRT performance. While an exploratory within-condition analysis showed a concomitant increase in α-band IMC in a postural muscle pair (serratus anterior–lumbar multifidus), the absence of a significant Group × Time interaction prevents attributing the behavioral improvement to this specific neuromuscular change. Given the small, homogeneous sample (nine healthy young Japanese males) and the exploratory design, these results are hypothesis-generating and require replication in larger, adequately powered studies before any conclusions about underlying mechanisms or clinical application can be drawn.

## 1. Introduction

The olfactory system, one of the most ancient structures phylogenetically, is intricately linked to the limbic system, which plays a key role in memory and emotion. This system also connects to the subcortical control of olfactory–motor function [1]. Research on the olfactory–motor system has identified various responses, such as respiratory and eye movements in response to high concentrations of odor stimuli [2], a decrease in the output of single muscles following unpleasant odor stimuli [3], and the startle reflex to sound stimuli post-exposure to unpleasant odors [4]. These responses are understood in the context of active exploratory behavior associated with olfaction [5].

Studies have also investigated the relationship between olfaction and the motor system, particularly with food-related odor stimuli. Such stimuli have been found to influence swallowing function, postural stability, and gait performance in elderly populations [6,7,8,9], as well as standing balance and upper limb motor function in healthy adults and children [10,11]. Neuroimaging research indicates that food-related odors strongly activate brain reward circuits [12,13,14], with higher activation observed in the anterior cingulate cortex, insula, and putamen in response to food odors compared to non-food odors [15]. While the precise neuronal mechanisms underpinning the olfactory–motor relationship remain elusive, it is hypothesized that the activation of these brain regions by food odors may influence motor systems. To quantify the neural drive underlying such motor responses, intermuscular coherence (IMC) analysis—a method for evaluating oscillatory neural inputs shared between muscles based on surface electromyography (EMG) signals—provides a valuable tool for examining sensorimotor integration [16,17]. IMC reflects the extent to which coordinated muscle activity is mediated by common oscillatory neural drives, and has been applied to the study of postural control and upper limb motor coordination [18,19,20,21].

Food odors, directly linked to eating behavior, involve movements such as reaching with the upper limb and bending the trunk towards food. To date, the effects of odor stimuli on the activity of upper limb–trunk muscles in humans have not been thoroughly investigated. Therefore, our aim is to explain the neural underpinnings of the olfactory–motor system by examining muscle activity changes in upper limb–trunk movements induced by soy sauce odor as a potential motivator for eating. In our unpublished clinical observations during rehabilitation of patients with central nervous system disorders, exposure to food odors during cooking appeared to be associated with improvements in physical movements, including upper limb movements and standing balance; although anecdotal, this prompted the present investigation. Building on this, we reported that exposure to soy sauce odor, as opposed to β-phenylethyl alcohol (PEA; the primary component of rose odor) or water (odorless), improved performance in the modified functional reach test (mFRT), a dependable measure of sitting balance during upper limb extension [22].

To further explore the neuromuscular mechanisms underlying this phenomenon, the current study used EMG to record muscle activity during the mFRT before and after exposure to three different odor stimuli: soy sauce, PEA, and water. EMG recordings were taken from four muscles crucial in the anterior motion of the upper limb during the mFRT: the anterior deltoid (AD), upper trapezius (UT), serratus anterior (SA), and lumbar multifidus (LM). The scapula is thought to play an important role in optimizing upper limb function, providing a functional connection to the trunk and dynamic stability during upper limb activities [23,24]. Previous EMG studies targeting upper limb movements have primarily focused on the AD, UT, and SA [25,26,27]. The SA is essential for maximizing upper extremity reach, facilitating anterior extension and upward rotation of the scapula through its functional coupling with the trunk [28,29]. Additionally, the LM has been shown to precede AD activity in shoulder flexion movements and contribute to trunk stability during overall reaching activities [30,31,32]. Although the LM contributes to anticipatory postural adjustments [30,31] preceding limb movement, the present study examined intermuscular coordination during the reaching movement itself rather than anticipatory pre-activation. Therefore, we investigated the temporal changes in the activity of these four muscles during the mFRT.

The EMG data were analyzed for IMC between pairs of muscles. IMC assesses the linear correlation of specific frequencies between two EMG signals, evaluating the extent to which muscle activities are coordinated by oscillatory neural drives shared among multiple muscles during motor control [16,17]. For instance, α-band IMC increases during dynamic coordination tasks and slow movements, whereas β-band IMC decreases with increased static muscle contraction [33,34]. These variations may indicate the ability of the nervous system to alter the frequency of neural drive in response to different tasks or environmental conditions. Using these metrics, we examined the hypothesis that soy sauce odor can modulate the coordination and output of skeletal muscles involved in motor control.

## 2. Materials and Methods

### 2.1. Study Design

This study used a within-subject, three-period, three-sequence crossover design (reported in accordance with the CONSORT extension for crossover trials [35]; Supplementary Materials Table S1 CONSORT Checklist), where subjects received each of the three interventions with distinct odor stimuli (soy sauce, PEA, and water) in a randomized sequence over three consecutive experimental periods.

To ensure balanced allocation across all possible presentation orders, a balanced complete crossover scheme was employed: all six possible sequences of the three odor conditions were enumerated a priori, and two participants were randomly assigned to each of the six sequences, such that all twelve recruited participants were allocated across the complete set of sequences [36]. The allocation sequence was generated by the principal investigator (Y.Y.), and participants were blinded to odor identity; however, the experimenter administering the odor was aware of the assigned condition (open-label for the experimenter).

A washout period of at least 1 day (range: 1–83 days; mean: 19.6 days; median: 3 days) was maintained between each period. The majority of intervals were short (≤10 days; mean: 2.1 days; *n* = 12 of 18 intervals); six intervals were extended (mean: 54.5 days) due to participants’ personal scheduling constraints unrelated to the study protocol. The washout interval was considered sufficient given that olfactory adaptation to a single brief exposure (20 s) resolves rapidly, typically within minutes to hours [37,38].

**Figure 1:**
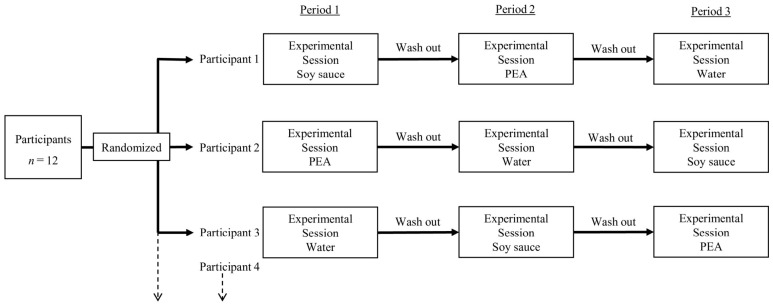
Study design: Three-sequence-crossover study. Participants received three different odor stimuli in a randomized order across three consecutive experimental periods, with ≥1 day as a washout period between each session.

The sample size for the study was determined using G* Power 3.1.9.7 software (Heinrich Heine University, Düsseldorf, Germany). A linear mixed model (LMM) and analysis of variance (ANOVA) were applied to test the effects of the three interventions, using a 3 × 3 crossover design. Based on our previous study [22] demonstrating a large effect size (*r* = 0.778, *z* = 3.814), we adopted the standard criterion for a “large” effect (*f* = 0.40) proposed by Cohen [39]. With an α error probability of 0.05 and a power (1-β error probability) of 0.8, and a conservative correlation among repeated measures of 0.70, the required sample size was calculated to be nine participants. Initially, twelve participants were recruited to account for potential dropouts (Figure 1). Detailed effect sizes from previous studies used for these calculations are provided in Supplementary Materials Table S2.

Because this sample size was determined a priori for the mFRT as the primary endpoint, the intermuscular coherence analyses should be regarded as exploratory; the study was not formally powered for the IMC outcomes. No formal power analysis was conducted for the IMC endpoints, given the 216 IMC values per participant across six muscle pairs, three time periods, two frequency bands, and three odor conditions. Accordingly, all IMC-related findings should be interpreted as hypothesis-generating rather than confirmatory.

The study received approval from the Research Ethics Committee at Tosa Rehabilitation College (TRC; registration No. TRC201801). All participants provided informed consent, in accordance with the Declaration of Helsinki. Written informed consent was obtained from all participants prior to their inclusion in the study, including consent for the publication of the research results.

### 2.2. Participants

Participants were recruited from the student body of TRC. They were informed about the study’s objectives and procedures through a written document and provided written informed consent. The study included twelve male participants. Of the twelve participants recruited, three were excluded from the final analysis: one withdrew due to scheduling conflicts, and two were excluded due to data loss in the electromyography (EMG) recordings.

**Figure 2:**
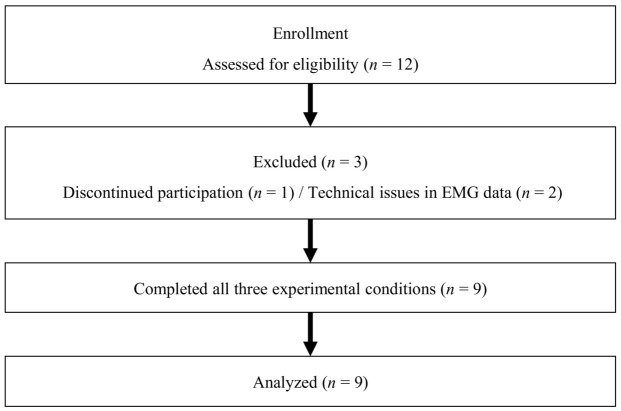
Participant flow diagram. Twelve subjects enrolled, three were excluded (one withdrawal, two EMG issues) and nine were analyzed.

Consequently, data from the remaining nine participants (mean age: 21.7 ± 1.8 years; mean weight: 66.8 ± 3.26 kg; mean height: 174.1 ± 1.73 cm) were included in the final statistical analysis (Figure 2). To assess potential systematic bias introduced by these exclusions, the baseline characteristics of the excluded participants (mean age: 21.7 ± 0.6 years; mean weight: 54.7 ± 5.5 kg; mean height: 167.3 ± 0.6 cm) were compared with those of the included participants (Table 1). Although the excluded participants tended to have lower body weight, no systematic differences in age or height were observed, and the exclusions were attributable to technical (EMG data loss) and scheduling reasons unrelated to participant characteristics or task performance.

To eliminate the potential influence of fluctuations in olfactory sensitivity associated with the menstrual cycle [40,41], only male participants were recruited. Olfactory sensitivity is known to fluctuate across the menstrual cycle, with detection thresholds varying systematically around the time of ovulation [40,41]. Further, significant sex differences in muscle activation patterns of the scapular region during functional upper-limb movements have been reported [42,43]. To minimize such physiological and kinematical variability, this study focused exclusively on a male cohort.

Participants were confirmed to be free from neuromusculoskeletal conditions affecting upper limb movement and from ear, nose, and throat disorders such as rhinitis, verified through an annual physical examination at TRC. In addition, none of the participants were smokers.

### 2.3. Task

#### 2.3.1. Experimental Setup

Participants were tested in a controlled environment at 26 °C. To ensure a fasted state of at least 4 h post-meal, participants were instructed to finish breakfast by 8:00 a.m. for the 12:00–2:00 p.m. sessions, or lunch by 1:00 p.m. for the 5:00–6:00 p.m. sessions. During the fasting period, only water was permitted, and the consumption of caffeine (e.g., coffee) and sugar-sweetened beverages was prohibited. Each experimental session included the presentation of one of the three odors, chosen randomly.

Participants were unaware of which odor would be used. mFRT and EMG measurements were taken both before and after odor exposure. A total of three experimental sessions were conducted for each participant, with ≥1 day between sessions.

The odors used were soy sauce (Honjozo Koikuchi Soy Sauce, Kikkoman Foods, Chiba, Japan), PEA (T&T Olfactometer panel selection standard concentration [A-4.0], Daiichi Pharmaceutical Industry, Tokyo, Japan), and water (Natural Water, Suntory, Tokyo, Japan). The exposure procedure involved applying 1 mL of the solution to odor paper (Daiichi Pharmaceutical Sangyo, Tokyo, Japan) and presenting it to the subjects for 20 s directly under the nasal cavity, approximately 1–2 cm from the nostrils. This 20-s exposure duration was adopted from our previous study, in which the same protocol produced a significant effect on mFRT performance [22].

Soy sauce was selected as the olfactory stimulus based on several considerations. Our previous study demonstrated that soy sauce odor significantly increases reaching distance in the mFRT [22]. Furthermore, food-related odors are known to activate the brain’s reward and dopaminergic circuits [13,15]. Cross-cultural research has also shown that soy sauce odor elicits high familiarity and pleasantness internationally [44], and is strongly perceived as an edible, appetizing odor, especially among Japanese individuals [45]. Consequently, soy sauce was chosen because the psychological and neural responses to its odor have been reported in previous studies. PEA, the primary odor component of rose oil, is frequently used in cosmetics, aromatherapy and human olfactory testing [46] and provides a pleasant and relaxing effect [47]. Water served as an odorless control.

#### 2.3.2. Subjective Evaluation of Odor Stimuli

After the entire sessions of mFRT, participants were asked impressions of the odor. The questionnaire included appetizing quality, familiarity, pleasantness, relaxing quality and intensity, which were rated on a 7-point scale from “very much agree” (+7) to “not at all agree” (+1) using the Likert method [45].

#### 2.3.3. mFRT

The mFRT is a well-established measure of sitting balance. This test is used for comparing the sitting balance of individuals with spinal cord injuries and strokes to healthy subjects [48,49,50], as well as for assessing the sitting balance performance across age groups: young, middle-aged, and older [51]. The mFRT reflects functional activities essential for daily living.

**Figure 3:**
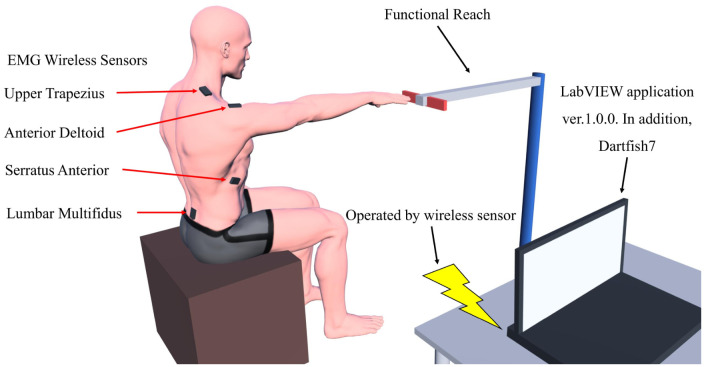
EMG wireless sensor placement and experimental setup. Four EMG wireless sensors were positioned on the upper trapezius (UT), anterior deltoid (AD), serratus anterior (SA), and lumbar multifidus (LM) muscles. The EMGs were recorded using the LabVIEW application and Dartfish7 software. Participants performed upper extremity reaching movements in response to cues given by the experimenter (see Figure 4).

During the experimental sessions, participants were seated in a chair with an FRT instrument aligned with the subject’s shoulder at the acromion height. mFRT scores were recorded using an FRT measurement device (GB-200, OG Wellness, Okayama, Japan). The starting position for the participants was as follows: lower limbs at 90° flexion at the hips, knees, and ankles; upper limbs at 90° shoulder flexion, elbow flexion, forearm extension, forearm rotation, wrist at midline, and hand extended. The distance from the tip of the hand in the starting position to the tip of the hand at maximum reach (FRT max) was measured in 1-mm increments (Figure 3).

**Figure 4:**
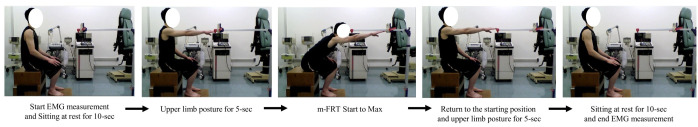
EMG measurement procedure. EMG recordings were conducted before and after odor exposure. The standardized procedure involved sitting at rest for 10 s, maintaining an upper limb movement posture for 5 s, performing the mFRT from FRT start to FRT max, returning to the starting position, holding the upper limb movement posture again for 5 s, and then sitting at rest for 10 s. This process was repeated following a 20-s exposure to the odor stimulus.

#### 2.3.4. Electromyograph Placement and Measurement Equipment

Electrode placement followed the SENIAM guidelines in the surface EMG manual [52]. Skin surfaces were cleaned with alcohol, and muscles were palpated before placing the electrodes parallel to the muscle fibers. We used four wireless electromyographs (LP-WS1221, Logical Products, Fukuoka, Japan), herein referred to as EMGs. These wireless sensors feature integrated bipolar electrodes with a fixed inter-electrode distance (20 mm), inherently complying with SENIAM recommendations. This configuration minimizes the risk of EMG crosstalk between adjacent muscles, while the wireless design eliminates cable-induced motion artifacts. Prior to measurements, we functionally verified isolated signal capture by performing specific joint movements. Signal integrity was strictly maintained through real-time visual monitoring and subsequent inspection of raw data to ensure the absence of artifacts or abnormal noise.

These EMGs were controlled by a personal computer (Versa Pro J-VF-F, NEC, Tokyo, Japan) equipped with a wireless sensor module ver. 7.5.5. LabVIEW application ver. 1.0.0. Dartfish7 software (Dartfish Inc., Tokyo, Japan) was installed on the same computer and synchronized with the EMG to capture a video of the measurement period. The computer was not connected to the internet during measurements. EMG data were recorded at a sampling frequency of 1000 Hz and subsequently stored on the computer using a parallel data acquisition application (LP-SUAP02-01, Logical Product Co., Ltd., Tokamachi, Japan) after measurement (Figure 3).

#### 2.3.5. Measurement Procedure

**Figure 5:**
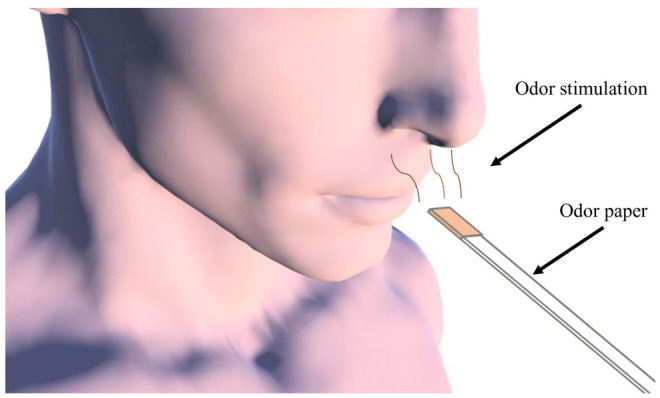
Olfactory Stimulation Method. During odor stimulation, odor paper was positioned directly beneath the participant’s nasal cavity for 20 s. Subjects were not informed about the specific odor presented.

To minimize variability among subjects, the EMG recording procedure before and after odor exposure was standardized as follows: (1) sitting at rest for 10 s, (2) maintaining the upper limb movement posture for 5 s, (3) starting mFRT, (4) reaching mFRT max, (5) returning to the starting position, (6) holding the upper limb movement posture again for 5 s, and (7) sitting at rest for 10 s. Following a 20-s exposure to the odor stimulus, the EMG measurement was repeated using the same steps (1) to (7). This entire process, including task guidance and EMG measurement, was continuously monitored by the operator using the recording screen of the video camera for accuracy and consistency (Figure 4 and Figure 5). The mFRT was measured only once before and after the odor stimulus to avoid performance improvement from repeated execution [53].

This methodology is consistent with the mFRT measurement procedure described in our previous study [22]. The 20-s odor exposure duration was chosen based on the physiological response duration and adaptation time for odor stimulation [37,38].

### 2.4. Data Analysis

#### 2.4.1. Analysis Time

**Figure 6:**
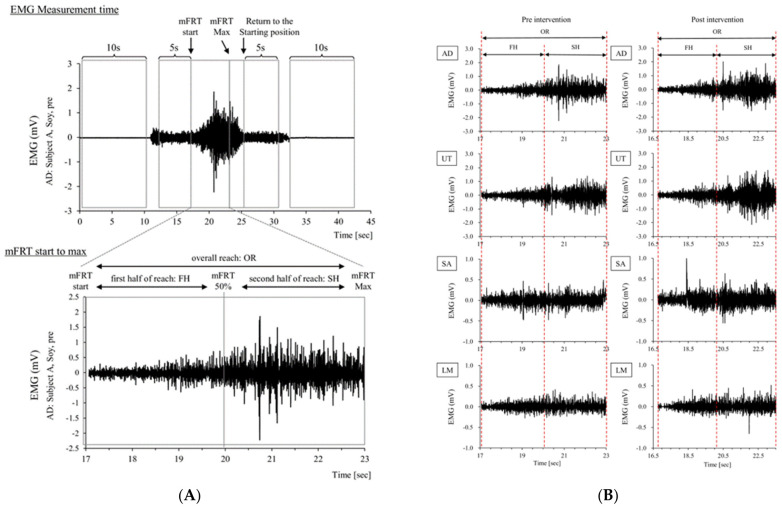
Analysis time periods of mFRT and EMG, Raw data recorded from four muscles. (**A**) The EMG analysis results of the AD muscle during the mFRT. The entire EMG analysis period, from FRT start to FRT max of the mFRT, is defined as the OR, which is subdivided into two phases: FH and SH. (**B**) EMG recordings from the AD, UT, SA, and LM muscles during the mFRT (from FRT start to FRT max), both before (pre) and after (post) exposure to soy sauce odor. The left panels represent pre-stimulation data, and the right panels show post-stimulation data. OR, overall reach; FH, first half of the reach; SH, second half of the reach. The red dashed line indicates FRT 50%, the midpoint dividing the first and second halves of the reach.

The surface EMG data collected during the mFRT were analyzed by segmenting the reach period into two distinct phases. Throughout the mFRT, shoulder flexion transitions from 90° to 180°. The muscle activity of the SA typically reaches 80–85% of its maximum strength at shoulder flexion angles between 120° and 160° [54,55]. Additionally, the LM muscle exhibits significantly higher activity towards the final range of the FRT movement compared to the initial range [56]. Based on these observations of muscle activity increments, the time from the start of FRT (FRT start) to FRT max was divided into two equal periods at the midpoint (FRT 50%). This division resulted in the first half of the reach (FH; FRT start − FRT 50%) and the second half of the reach (SH; FRT 50% − FRT max). IMC was analyzed during the FH, SH, and overall reach period (OR; FRT start − FRT max). EMG data from one subject during the reach period are presented in Figure 6A, and those for a single experimental session from one subject are shown in Figure 6B.

#### 2.4.2. Analysis Method

EMG data for each subject were sampled at 1000 Hz, then trimmed and preprocessed using MATLAB R2019a (MathWorks, Natick, MA, USA) and Origin 2022b (OriginLab, Northampton, MA, USA) based on the established analysis time. During the data collection and analysis, a visual inspection was conducted on the raw EMG recordings to confirm that the raw data did not exhibit any artifacts, noise, or EMG crosstalk. The wireless EMG sensors (LP-WS1221) incorporate an internal analogue bandpass filter of 19.6–442 Hz, which attenuated low-frequency motion artifacts and high-frequency noise at the hardware level prior to digital recording, in line with SENIAM recommendations [57]. The trimmed raw data were subsequently processed with a fourth-order zero-phase Butterworth low-pass filter (cutoff: 350 Hz) to further attenuate residual high-frequency noise. Full-wave rectification was not applied, as this nonlinear transformation is known to distort coherence estimates in the α- and β-bands [58,59,60].

The EMG of each muscle pair was transformed into the frequency domain, and IMC estimates were calculated using a 1024-point fast Fourier transform with a 1024-point Hamming window and 50% overlap. The 1024-point FFT provided a frequency resolution of approximately 0.98 Hz, which was considered sufficient for evaluating the spectral characteristics of the targeted oscillations.

Six muscle pair patterns were examined: SA–LM, SA–AD, SA–UT, LM–UT, LM–AD, and AD–UT. In total, the preprocessed EMG data for one participant involved analyzing 216 IMC values (1944 for all participants), considering six patterns of IMC before and after stimulation with three odors (soy sauce, PEA, and water), across three time periods (overall, first half, and second half of mFRT), and frequency bands of the α- and β-bands.

Signal quality was assessed using root-mean-squared (RMS)-based signal-to-noise ratio (SNR), calculated as 20·log_10_ (RMS task/RMS rest), where RMS rest was derived from a 10-s pre-task resting period in accordance with SENIAM recommendations. Grand-mean SNR across all muscles, conditions, and subjects was AD: 21.4 ± 8.4 dB, UT: 27.5 ± 8.0 dB, SA: 24.6 ± 8.4 dB, LM: 16.9 ± 6.6 dB, confirming acceptable signal quality for all channels.

#### 2.4.3. Frequency Bands

Oscillations in the α-band are observed in the activity of various muscles during unilateral and bimanual motor control tasks in both upper and lower limbs [18,19], as well as during stable movements where α-band IMC is prominent [20,21]. The 8–14 Hz frequency band, commonly used in electroencephalogram (EEG) analysis [61], was chosen for α-band IMC analysis. β-band activity, associated with independent muscle control rather than coordination, often increases during tasks such as pinch-grip, improving synergistic activity in hand muscles [34,62]. For β-band IMC analysis, the 15–35 Hz frequency band, frequently studied in β-band IMC research [63,64], was selected.

Although coherence in the θ-band (4–7 Hz) is primarily associated with postural control during quiet standing [19,20,65] or pathological tremors (e.g., essential tremor or dystonic tremor) [66], and γ-band (>35 Hz) activity typically emerges during higher-force or rapid movement conditions rather than slow voluntary movements [33], the functional reaching task (mFRT) utilized in the present study involves smooth and slow voluntary movements. Furthermore, task-related θ-band IMC has not been consistently reported in the proximal upper limb and trunk muscles targeted in the present study during voluntary reaching. Consequently, we focused on the α- and β-bands as the primary frequency ranges of interest for evaluating task-related intermuscular coordination during the mFRT, based on prior literature and the smooth, slow voluntary nature of the task.

### 2.5. Statistical Analysis

#### 2.5.1. Subjective Evaluation of Odors and mFRT

Subjective ratings of odors were statistically compared among three odors using Friedman test and between two odors using Wilcoxon signed rank sum test with Bonferroni correction. The normality of the mFRT score data was confirmed using the Shapiro–Wilk test. To verify baseline equivalence of mFRT scores across the three odor conditions prior to intervention, pre-odor scores were compared using a one-way repeated measures ANOVA. To assess carry-over effects across experimental periods, pre-intervention mFRT scores were compared across the three periods (Period 1, 2, and 3, corresponding to chronological session order). Normality was assessed using the Shapiro–Wilk test, and a one-way repeated-measures ANOVA or Friedman test was applied accordingly. A two-way repeated measures ANOVA, with a post-hoc Bonferroni test for multiple comparisons, was conducted to analyze the changes in mFRT scores before and after odor stimulation.

This analysis compared the scores before and after stimulation, across the three groups, and examined the interaction term. Statistical analysis was conducted using SPSS software (ver. 28.0; IBM Corp., Armonk, NY, USA). The statistical significance was set at *p* < 0.05.

#### 2.5.2. IMC in the α- and β-Bands

The coherence function, used to measure the correlation between two signals [67], was used in the analysis of α- and β-band IMC values for the six muscle pairs of all participants. These analyses were performed using Stata ver. 17 (Stata Corp LLC, College Station, TX, USA). The coherence function is defined by the equation:

$${Coh}_{xy}(f)=\frac{|P_{xy}(f){|}^{2}}{P_{xx}(f)P_{yy}(f)}$$

where ${Coh}_{xy}\left(f\right)$ is the coherence function of signals *x* and *y* at frequency f, $P_{xy}(f)$ is the cross spectrum of signals x and y, and $P_{xx}(f)$ and $P_{yy}(f)$ are the power spectra of signals x and y, respectively. Due to the scale of both IMC values and peak cross-correlation coefficients (range: 0–1), Fisher’s z-transformation was applied to normalize these data for statistical comparisons across participants and tasks. This transformation adjusts the distribution of coherence values to be closer to a normal distribution, aiding in statistical testing and estimation [17]. Specifically, this transformation was employed to achieve variance stabilization of the coherence estimates, ensuring the data met the assumptions required for parametric statistical testing.

In the following formula, n is the number of overlapping spectral segments, which varied across participants and analysis time periods according to individual reach duration (OR: range 6–16, mean 9.74; FH and SH: range 3–8, mean 4.61), calculated using the standard 1024-ms window with 512-ms (50%) overlap described in Section 2.4.2, except for two sessions, in which the FH period was shorter than 2048 ms and a shorter window (512 ms) with reduced overlap (256 ms) was applied accordingly. $coh\left(f\right)$ is the coherence of the corresponding frequency.

$$z=\sqrt{\left(2n\right)}*Tanh^{-1}\sqrt{coh\left(f\right)}$$

An LMM analysis was used to evaluate changes in IMC (IMCΔ) before and after exposure to the three odor stimuli, in relation to the calculated IMC (Fisher-*z*) in the α- and β-bands. In this model, subjects were treated as a random factor, while the three odor stimulus groups (soy sauce, PEA, and water), time (before and after odor stimulation), and their interaction terms were treated as fixed factors. The model allowed for a before/after comparison within each stimulus group, and a comparison of the differences in change before and after the three odor stimuli (interaction term). The LMM analysis provided least-squares means and 95% confidence intervals as estimates of the IMC (Fisher-*z*) for each condition (analysis time, two frequencies, and muscle pair). The statistical significance was set at *p* < 0.05. To control the false discovery rate (FDR) across the large number of post-hoc pairwise comparisons, the Benjamini–Hochberg (BH) procedure was applied separately within each frequency band (α and β; 54 comparisons each) [68].

## 3. Results

### 3.1. Subjective Evaluation of Odors

**Figure 7:**
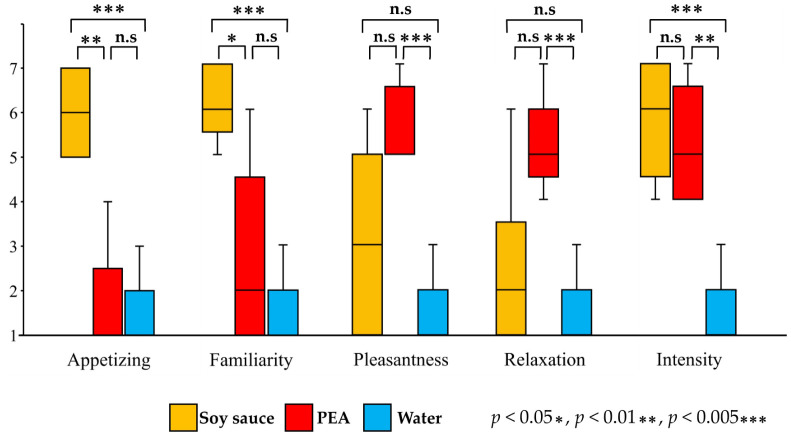
Subjective evaluation of odors. Odors were subjectively evaluated for features as indicated, by rating from +7 to +1. Soy sauce (orange), PEA (red), and water (blue). Boxes represent the 25th and 75th percentiles, whiskers indicate minimum and maximum values, lines inside boxes represent medians. n.s., not significant.

The values among three different odors (soy sauce, PEA, water) were statistically different in all features examined (appetizing, familiarity, pleasantness, relaxation, intensity) (Friedman test; Supplementary Materials Table S3). In the comparison between two odor pairs, odor of soy sauce was rated significantly higher than PEA and water in appetizing and familiarity (Wilcoxon signed rank sum test with Bonferroni correction; Figure 7, and Supplementary Materials Table S4 for *p* values).

Odor of PEA was rated significantly higher than water in pleasantness and relaxation. Odor of soy sauce seemed to be rated higher than water and lower than PEA in pleasantness and relaxation, but these were not statistically significant. Both soy sauce and PEA odors were rated significantly higher than water in intensity. Intensity rating of soy sauce and PEA odors was not significantly different (Supplementary Materials Table S4 for *p* values).

Regarding odor identity and imagery, odor of soy sauce reminded the participants of seasonings (soy sauce and buttery soy sauce odor) and related foods (stir-fried noodles and sushi). Odor of PEA reminded them of perfume and air freshener. No participant claimed that they felt uncomfortable with the odor stimuli.

Participants felt odorless for water presentation. In the subjective evaluation of odors, three participants rated the intensity of the water condition above 1 (scores of 2–3 on a 7-point scale), because they felt it odorless but speculated that it might have some odor (participants were not informed of which odor would be used). Their free-text responses confirmed that none identified a specific odor (e.g., “I felt nothing, but thought something might be there”), indicating that these ratings reflected ambiguity rather than genuine odor detection. As a blinding check, odor intensity ratings confirmed that water was rated significantly lower than both soy sauce and PEA (*p* = 0.003 and *p* = 0.007, respectively; Supplementary Materials Table S4), whereas soy sauce and PEA did not differ in intensity (*p* = 1.00), indicating that participants could not discriminate the water control as a distinctive odor.

Taken these results together, soy sauce and PEA odors were similar in intensity and mostly favorable for participants, and soy sauce odor was more food-related, appetitive and familiar than PEA odor.

### 3.2. mFRT

The Shapiro–Wilk test indicated non-normality for Period 3 (W = 0.828, *p* = 0.043) (Table 2); therefore, a Friedman test was applied. Pre-intervention mFRT scores were stable across the three periods, and no significant period effect was detected (χ^2^ (2) = 0.74, *p* = 0.690; Kendall’s W = 0.04), confirming that baseline motor performance was not systematically influenced by session order and that carry-over effects of odor exposure were negligible.

Table 3 presents the two-way ANOVA results of the reach distances measured in the mFRT before and after exposure to the three odor stimuli. There was a significant interaction effect observed between the groups and the before/after change (*F* [2, 24] = 3.70, *p* = 0.04). While there was no significant difference in the main effect among the three groups (*F* [2, 24] = 0.12, *p* = 0.887), a notable difference was found in the before/after comparison (*F* [2, 24] = 6.23, *p* = 0.020). Post-hoc Bonferroni analysis indicated a significant increase in the mFRT score after exposure to soy sauce odor compared to before exposure (*p* < 0.001), but no significant differences were observed for the PEA or water odors (PEA: *p* = 0.763, water: *p* = 0.725). Within-session test–retest reliability [69] of the mFRT, assessed using the water control condition, was excellent [ICC (3,1) = 0.965, 95% CI: 0.840–0.991; Table 4]. In contrast, ICC was not calculated for IMC, as this measure fluctuated even under the water control condition, which is inconsistent with the assumption of a stable measurement underlying test–retest reliability [69].

### 3.3. IMC Comparisons at Each Time Point

The IMC values before and after olfactory stimulation, along with between-group comparisons at each time point, are detailed in the Supplementary Materials (Figure S1; α-band and β-band IMC for all muscle pairs with BH-corrected *p*-values and effect sizes, and Supplementary Table S5 for the full unadjusted and BH-adjusted *p* values for all comparisons). All *p*-values reported below are Benjamini–Hochberg (BH) corrected for multiple comparisons. Effect sizes are reported as Cohen’s *d*.

#### 3.3.1. α-Band IMC

**Figure 8:**
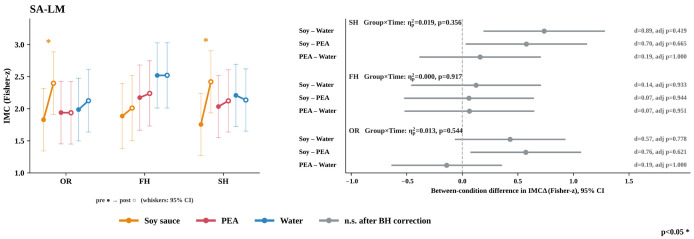
Alpha-band (8–14 Hz) intermuscular coherence (IMC) of the SA–LM muscle pair pre and post olfactory stimulation. (**Left** panel) IMC values (Fisher-*z*) before (pre, filled circles) and after (post, open circles) olfactory stimulation for the overall reach (OR), first half (FH), and second half (SH) of the mFRT. Whiskers indicate 95% confidence intervals. Asterisks indicate significant within-condition increases following soy sauce exposure (BH-corrected). (**Right** panel) Between-condition differences in ΔIMC (Fisher-*z*) with 95% CIs. All comparisons are non-significant after BH correction (shown in gray). SA, serratus anterior; LM, lumbar multifidus; OR, overall reach; FH, first half of reach; SH, second half of reach. * *p* < 0.05.

Within-condition analyses of the SA–LM pair revealed significant increases in α-band IMC following soy sauce exposure at OR (*d* = 1.07, adjusted *p* = 0.039; Figure 8: SA–LM, left panel) and SH (*d* = 1.13, adjusted *p* = 0.038). No significant within-condition changes were observed after PEA or water exposure at any time period (all adjusted *p* > 0.05). The Group × Time interaction was not significant for the SA–LM pair at any time period (all adjusted *p* > 0.05; Figure 8: SA–LM, right panel). No significant within-condition changes or Group × Time interactions were observed in any other muscle pair (AD–SA, SA–UT, LM–UT, LM–AD, or AD–UT) at any time period (all adjusted *p* > 0.05; Figure S1). These results indicate that soy sauce odor selectively increased α-band IMC between the SA and LM muscles, whereas no significant between-condition differences were observed after correction.

#### 3.3.2. β-Band IMC

**Figure 9:**
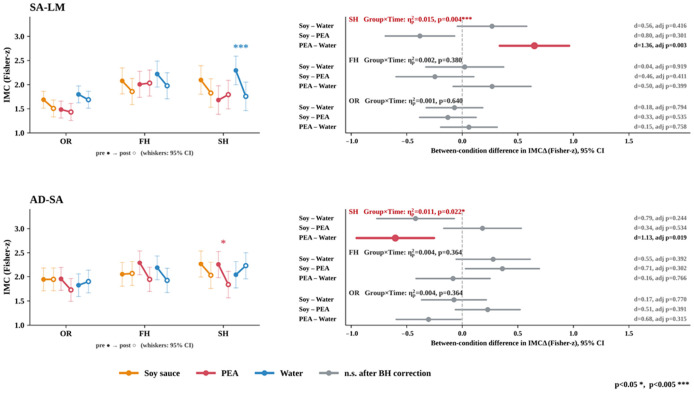
Beta-band (15–35 Hz) intermuscular coherence (IMC) of the SA–LM and SA–AD muscle pairs pre and post olfactory stimulation. (Upper panels: SA–LM; Lower panels: SA–AD) **Left** panels: IMC values (Fisher-*z*) before (pre, filled circles) and after (post, open circles) olfactory stimulation at OR, FH, and SH. Whiskers indicate 95% confidence intervals. **Right** panels: Between-condition differences in ΔIMC (Fisher-*z*) with 95% CIs. Red lines indicate significant Group × Time interactions after BH correction; gray lines are non-significant. SA, serratus anterior; LM, lumbar multifidus; AD, anterior deltoid; OR, overall reach; FH, first half of reach; SH, second half of reach. * *p* < 0.05, *** *p* < 0.005.

For the SA–LM pair, a significant Group × Time interaction was observed during SH (partial η^2^_p_ = 0.015, adjusted *p* = 0.004; Figure 9: SA–LM, right panel). Post-hoc comparison of IMC change ratios revealed that water exposure produced a significantly greater reduction than PEA exposure during SH (*d* = 1.36, adjusted *p* = 0.003). Within-condition analyses revealed a significant decrease following water exposure at SH (*d* = 1.59, adjusted *p* < 0.001; Figure 9: SA-LM, left panel). No other significant within-condition changes were observed for this pair at any time period (adjusted *p* > 0.05).

For the AD-SA pair, a significant Group × Time interaction was observed during SH (partial η^2^_p_ = 0.011, adjusted *p* = 0.022; Figure 9: AD-SA, right panel). Post-hoc comparison of IMC change ratios revealed that PEA exposure produced a significantly greater reduction than water exposure during SH (*d* = 1.13, adjusted *p* = 0.019). Within-condition analyses revealed a significant decrease following PEA exposure at SH (*d* = 1.10, adjusted *p* = 0.026; Figure 9: AD-SA, left panel). No other significant within-condition changes were observed for this pair at any time period (adjusted *p* > 0.05).

No significant Group × Time interactions or within-condition changes were observed in the remaining muscle pairs (SA–UT, LM–UT, LM–AD, or AD–UT) at any time period (all adjusted *p* > 0.05; Figure S1).

## 4. Discussion

In this exploratory study, exposure to soy sauce odor, but not to PEA or water odors, led to an increase in the reach distance during the mFRT. EMG analysis showed an increase in α-band IMC between the SA and LM muscles following exposure to soy sauce odor. Because this within-condition increase was not accompanied by a significant Group × Time interaction, it should be regarded as a preliminary, hypothesis-generating observation rather than evidence that the behavioral improvement was caused by coordinated activation of these muscles. The selective pattern of IMC increase, confined to a single muscle pair and a single odor condition, represents the primary neurophysiological finding of this exploratory study and is discussed below in the context of existing literature on IMC during voluntary motor tasks.

### 4.1. α-Band IMC

In this exploratory study, within-condition analysis revealed that α-band IMC between the SA–LM muscle pair increased significantly following soy sauce odor exposure during the overall reach period and the second half of the reach after applying the BH correction for multiple comparisons, while Group × Time interaction for the SA–LM pair was not statistically significant at any time period after the correction. No significant within-condition changes were observed following PEA or water exposure at any time period. No significant within-condition changes or Group × Time interactions were observed in any of the remaining five muscle pairs across any time period or frequency band. These observations showed the selective property of α-band IMC increase, confined to a single muscle pair and a single odor condition in within-condition comparisons.

Alpha-band oscillatory activity (8–14 Hz) in the neuromuscular system has been associated with slow voluntary movements [70] and with postural control mechanisms [71], and increases in α-band IMC between limb muscles have been reported during low-speed dynamic and bimanual motor tasks [62,72,73,74]. The SA contributes to scapular forward projection and upward rotation, facilitating maximal reach distance [28,29], while the LM contributes to trunk stability during reaching movements [30,31,32]. SA muscle activity has been reported to approach near-maximal levels at shoulder flexion angles of approximately 120–160° [54,55], and LM activity has been shown to be higher during the later phase of the functional reach [56]. These functional characteristics may help to explain why the within-condition increase in α-band IMC was most consistently observed during the second half of the reach period in the present study. The SA and LM have been studied in the context of upper limb–trunk coordination, with evidence that scapular stabilizer activity and paraspinal muscle activity are modulated in relation to each other during upper limb tasks [75,76]. The selective within-condition increase in α-band IMC in the SA–LM pair following soy sauce odor exposure is therefore consistent with the possibility that this odor may have contributed to the coordinated activation of these postural muscles during the latter phase of the reaching movement. As only a single pre- and post-odor measurement was performed per condition, and given the small sample size of this exploratory study, we consider that this interpretation requires replication in larger, appropriately powered studies.

Regarding the neural origins of α-band EMG coherence, subcortical pathways—including brainstem structures and spinal interneuronal networks—have been suggested as one possible source of α-frequency oscillatory inputs to spinal motoneurons [74,77]; In addition, α-band oscillations are also known to be influenced by sensorimotor cortical activity [61], while the relative contributions of cortical and subcortical sources during voluntary upper limb movements have not been fully characterized. The SA and LM are proximal, postural muscles regulated in part by the medial motor control system, including the subcortical reticulospinal tract [78,79]. The observation that the within-condition α-band IMC increase was confined to this postural muscle pair, rather than to the more distal muscle pairs also examined, may reflect some degree of pathway specificity in the olfactory–motor relationship. Elucidating this hypothetical mechanism will require future studies incorporating direct measures of brain activity, such as combined EEG–EMG recording.

### 4.2. β-Band IMC

In this exploratory study, β-band IMC between the SA–LM muscle pair decreased significantly following water odor exposure during the second half of the reach, and water exposure produced a significantly greater reduction than PEA exposure during the period. β-band IMC between the AD–SA muscle pair decreased significantly following PEA odor exposure during the second half of the reach, and PEA exposure produced a significantly greater reduction than water exposure during the period. No other significant differences in within-condition or between-condition comparisons were observed in any odor exposure for any muscle pair.

The non-specific nature of this pattern—observed regardless of odor type—indicates that it is unlikely to reflect odor-specific neural changes. Notably, this finding stands in functional contrast to the soy sauce–specific within-condition increase in α-band IMC in the SA–LM pair. This frequency-specific dissociation may itself be informative: the observation that β-band IMC tended to decrease in different conditions, while α-band IMC in the SA–LM pair showed a selective within-condition increase only following soy sauce odor exposure, argues against the possibility that the α-band result reflects a non-specific adaptation effect common to all conditions.

The non-specific reduction in β-band IMC is consistent with well-established movement-related β desynchronization, whereby β-band oscillatory activity decreases during the preparation and execution of voluntary movements [80,81]. β-band IMC has also been reported to be higher during static isometric contractions than during dynamic motor tasks [33,34], and these observations together suggest that the reduction in β-band IMC in the present study may reflect a task-related rather than odor-specific phenomenon, including condition-dependent differences in attentional or arousal state rather than a direct olfactory effect on neuromuscular coordination.

The precise mechanism underlying this non-specific reduction in β-band IMC cannot be determined from the present data. The relationship between β-band IMC changes and mFRT performance was not formally assessed in the present study. Quantitative evaluation of respiratory activity or sniffing behavior, and other potential contributors such as condition-dependent differences in muscle activation levels or attentional state are required in future studies.

### 4.3. Methodological Considerations

Full-wave rectification of surface EMG signals prior to coherence analysis has been shown to introduce artificial low-frequency spectral components that can distort coherence estimates, particularly in the α and lower frequency bands [58,59,60]. To minimize this potential source of bias, the present study employed an unrectified analysis pipeline throughout, focusing on the α- and β-frequency bands considered most relevant to the voluntary sensorimotor task under investigation. Because the same unrectified pipeline was applied consistently across all experimental conditions, systematic preprocessing biases are unlikely to account for the condition-specific pattern observed selectively following soy sauce odor exposure. Nevertheless, it should be acknowledged that condition-dependent differences in EMG signal amplitude or spectral content may in principle exert differential influences on coherence estimates even when an identical processing pipeline is applied [58,59,60]. The absence of a direct comparison between rectified and unrectified coherence estimates for the SA–LM muscle pair remains a methodological limitation of the present study.

Beyond the rectification issue, the most significant methodological limitation of this study is its small sample size (*n* = 9). The sample size was determined a priori based on a large effect size for the mFRT primary outcome (*f* = 0.40); the IMC analyses were therefore not formally powered and should be regarded as exploratory. The small sample limits statistical power for detecting modest Group × Time interaction effects in the linear mixed model, and the absence of a statistically significant interaction for the SA–LM α-band pair after BH correction should be interpreted accordingly. These considerations are discussed further in Section 5.2.

## 5. Conclusions

### 5.1. Conclusions

This exploratory study examined the effects of olfactory stimulation on upper limb motor performance and neuromuscular coordination in nine healthy young Japanese males. Exposure to soy sauce odor, but not to PEA or water, produced a statistically significant increase in mFRT reach distance. Within-condition analyses revealed a selective increase in α-band IMC between the SA and LM muscles following soy sauce odor exposure, particularly during the second half of the reaching movement, while this pattern did not reach statistical significance in between-condition comparisons. The improvement in mFRT performance was statistically significant, whereas the increase in α-band IMC was a within-condition, exploratory observation that did not reach significance in between-condition (Group × Time) comparisons. The improvement in mFRT performance therefore cannot be attributed to this neuromuscular change. Given the small, homogeneous sample and the exploratory design, these findings are hypothesis-generating and require replication in larger, adequately powered studies before the odor effect is introduced into clinical or rehabilitative application.

### 5.2. Limitations and Future Research Considerations

The present study has several limitations that should be considered when interpreting its findings.

First, the participant cohort was restricted to healthy young Japanese males (mean age: 21.7 ± 1.8 years). Female participants were excluded owing to potential hormonal confounds on olfactory sensitivity across the menstrual cycle [40,41]. Furthermore, previous studies have reported significant sex differences in muscle activation patterns of the scapular region during functional upper-limb movements [42,43]. Such sex-related differences in neuromuscular recruitment patterns of the scapular musculature could constitute an additional source of kinematic variability. However, these exclusions limit the generalizability of the findings to other populations. Accordingly, future studies should include female and older participants to determine whether the present findings generalize across sex and age.

Second, the sample size (*n* = 9), determined a priori for the mFRT primary endpoint based on a large effect size from a prior study (*f* = 0.40), was insufficient to provide adequate statistical power for detecting the small Group × Time interaction effects observed in the IMC analyses (η^2^_p_ = 0.011–0.019). Post-hoc power analysis using a repeated-measures approximation (G*Power 3.1; *F*-test; α = 0.05; 3 conditions × 2 time points; numerator *df* = 2; inter-measurement correlation r = 0.50–0.70) indicated that approximately *n* = 27–74 participants would be required to detect these observed interaction effect sizes with 80% power in a single comparison. Estimated power at *n* = 9 was only 13–31% for these interaction effects. Accordingly, all IMC-related findings should be interpreted as preliminary and hypothesis-generating. By contrast, the large within-condition effect sizes for the SA–LM α-band pair following soy sauce exposure (Cohen’s *d* = 1.07–1.13) were detectable at the current sample size. The non-significant Group × Time interaction after BH correction may reflect insufficient power for between-condition comparisons at this sample size, rather than evidence of a true null effect. Relatedly, the IMC analyses involved a large number of comparisons (216 IMC values per participant across six muscle pairs, three reach periods, two frequency bands, and three odor conditions). The Benjamini–Hochberg procedure was applied within each frequency band to limit false-positive findings; nevertheless, given the large number of comparisons, the IMC findings should be interpreted as exploratory and hypothesis-generating.

Third, the washout period between experimental sessions was defined as ≥1 day (range: 1–83 days; mean: 19.6 days; median: 3 days), with the majority of intervals being short (≤10 days). While olfactory adaptation to a single brief exposure resolves rapidly [37,38], and no significant carry-over effect was detected in pre-intervention mFRT scores across periods (Table 2), the variable and sometimes short washout intervals may have introduced inconsistencies in hedonic or attentional states across sessions that could not be fully controlled. Future studies should employ a standardized washout period of sufficient duration to minimize potential carryover of odor-evoked affective or cognitive states.

Fourth, the present study was not registered in a clinical trial registry prior to data collection. Although the study was approved by the institutional ethics committee and conducted in accordance with the Declaration of Helsinki, the absence of prospective registration limits the verifiability of pre-specified hypotheses and outcomes. Future studies in this area should be prospectively registered.

Fifth, EMG recordings were limited to four muscles. Broadening future recordings to include bilateral trunk and lower extremity muscles would enable more comprehensive analysis of whole-body olfactory–motor coordination. Simultaneous EEG–EMG recording would also allow direct assessment of corticomuscular and corticospinal contributions to any observed IMC changes, and would help to distinguish cortical from subcortical neural generators—a distinction that cannot be made from the present peripheral EMG data alone [82].

Sixth, there was a methodological limitation regarding the evaluation of motor function. Although the mFRT is a practical and useful clinical measure, quantitative evaluations using posturography are required to elucidate the underlying postural control mechanisms in greater detail.

Finally, the absence of an objective, quantitative characterization of the olfactory stimulus, such as its concentration or standardized sensory evaluation, is a limitation that affects the reproducibility of the study. The odor stimuli were presented without a flow-controlled olfactometer, and odor concentration was not objectively measured. Although subjective intensity ratings did not differ significantly between soy sauce and PEA conditions, objective psychophysical controls would strengthen the rigor of olfactory stimulus delivery in future studies. In addition, the concentration-dependent effects of the odors were not examined, and future studies should investigate how varying stimulus intensities affect motor and neuromuscular outcomes. Comparative studies incorporating a broader spectrum of olfactory stimuli—including other food-related odors and malodors—would further clarify the specificity and neural basis of the effects observed in the present study.

## Figures and Tables

**Table 1:** Baseline characteristics of included and excluded participants.

	Included (*n* = 9)	Excluded (*n* = 3)
Age (years)	21.7 ± 1.8	21.7 ± 0.6
Weight (kg)	66.8 ± 3.3	54.7 ± 5.5
Height (cm)	174.1 ± 1.7	167.3 ± 0.6
Reason for exclusion	—	2 × EMG data loss; 1 × scheduling conflict

**Table 2:** Pre-intervention mFRT scores and carry-over analysis across experimental periods (*n* = 9).

	Period 1	Period 2	Period 3
Pre-mFRT, Mean ± SD (mm)	444.7 ± 59.2	454.7 ± 61.9	455.8 ± 65.9
Shapiro–Wilk, W (*p*)	0.987 (0.991)	0.950 (0.690)	0.828 (0.043) *
Friedman test	χ^2^ (2) = 0.74, *p* = 0.690 Kendall’s, *W* = 0.04		

* *p* < 0.05. Period 1–3: chronological session order, independent of odor assignment.

**Table 3:** mFRT scores before (pre) and after (post) olfactory stimulation.

Group	Pre-Odor	Post-Odor	*p* (Pre vs. Post)	Main Effect Group Comparison		Main Effect Pre-Post Comparison		Interaction	
				*F*	*p*	*F*	*p*	*F*	*p*
Intervention				0.120	0.887	6.238	0.020 *	3.705	0.040 *
Soy sauce	441.8 ± 66.8	465.8 ± 79.9	<0.001 ***						
PEA	449.4 ± 57.7	451.4 ± 60.9	0.763						
Water	463.7 ± 60.7	466.1 ± 69.5	0.725						

*, *p* < 0.05; ***, *p* < 0.005. Changes in mFRT scores assessed using two-way ANOVA, supplemented with post-hoc Bonferroni tests for multiple comparisons.

**Table 4:** Within-session test–retest reliability of the mFRT under the water control condition (*n* = 9).

Participant	Pre-mFRT (mm)	Post-mFRT (mm)	Difference (mm)
A	457	425	−32
B	514	524	+10
C	508	495	−13
D	393	384	−9
E	464	480	+16
F	348	348	0
G	543	570	+27
H	466	471	+5
I	481	498	+17
Mean ± SD	463.8 ± 60.7	466.1 ± 69.5	+2.3 ± 17.9
ICC (3,1) (95% CI)	0.965 (0.840–0.991)		
Interpretation	Excellent		

ICC: intraclass correlation coefficient (two-way mixed-effects model, absolute agreement, single measures); assessed following the model-selection guidelines of Koo & Li (2016) [69]. mFRT: modified functional reach test.

## Data Availability

The full trial protocol will be made available through the institutional repository of University of Kochi Health Sciences upon publication.

## Supplementary Materials

The following supporting information can be downloaded at: https://www.mdpi.com/article/10.3390/brainsci16070737/s1, Figure S1: Supplementary Data. Table S1: CONSORT Crossover checklist. Table S2: Effect Size Calculation from Previous Study. Table S3: Subjective evaluation of odors comparison among three odors. Table S4: Subjective evaluation of odors: comparison among two odors. Table S5: Cohen’s *d* for within-condition pre–post comparisons, partial η^2^_p_ for Group × Time interactions, and corresponding Benjamini–Hochberg (BH)-adjusted *p* values for intermuscular coherence (IMC).

## Abbreviations

The following abbreviations are used in this manuscript:

EMG	Electromyography
IMC	Intermuscular coherence
mFRT	Modified functional reach test
PEA	Phenylethyl alcohol
UT	Upper trapezius
AD	Anterior deltoid
SA	Serratus anterior
LM	Lumbar multifidus
