# Supplementary material for: Soy Sauce Odor Improves Upper Limb Motor Performance with Preliminary Evidence of Increased Alpha-Band Intermuscular Coherence Between Postural Muscles: An Exploratory Within-Subjects Crossover Study
Source: Brain Sci. 2026 Jul 12;16(7):737. doi: 10.3390/brainsci16070737 (PMC13407258; doi:10.3390/brainsci16070737)
Supplement: Supplementary file 1 [file brainsci-16-00737-s001.zip › brainsci-4265143-Figure S1. Supplementary Materials Data.pdf]

Supplementary Materials Data **Figure S1**

α-band (8–14 Hz) IMC — Linear mixed model, crossover design [df (2, 418)]

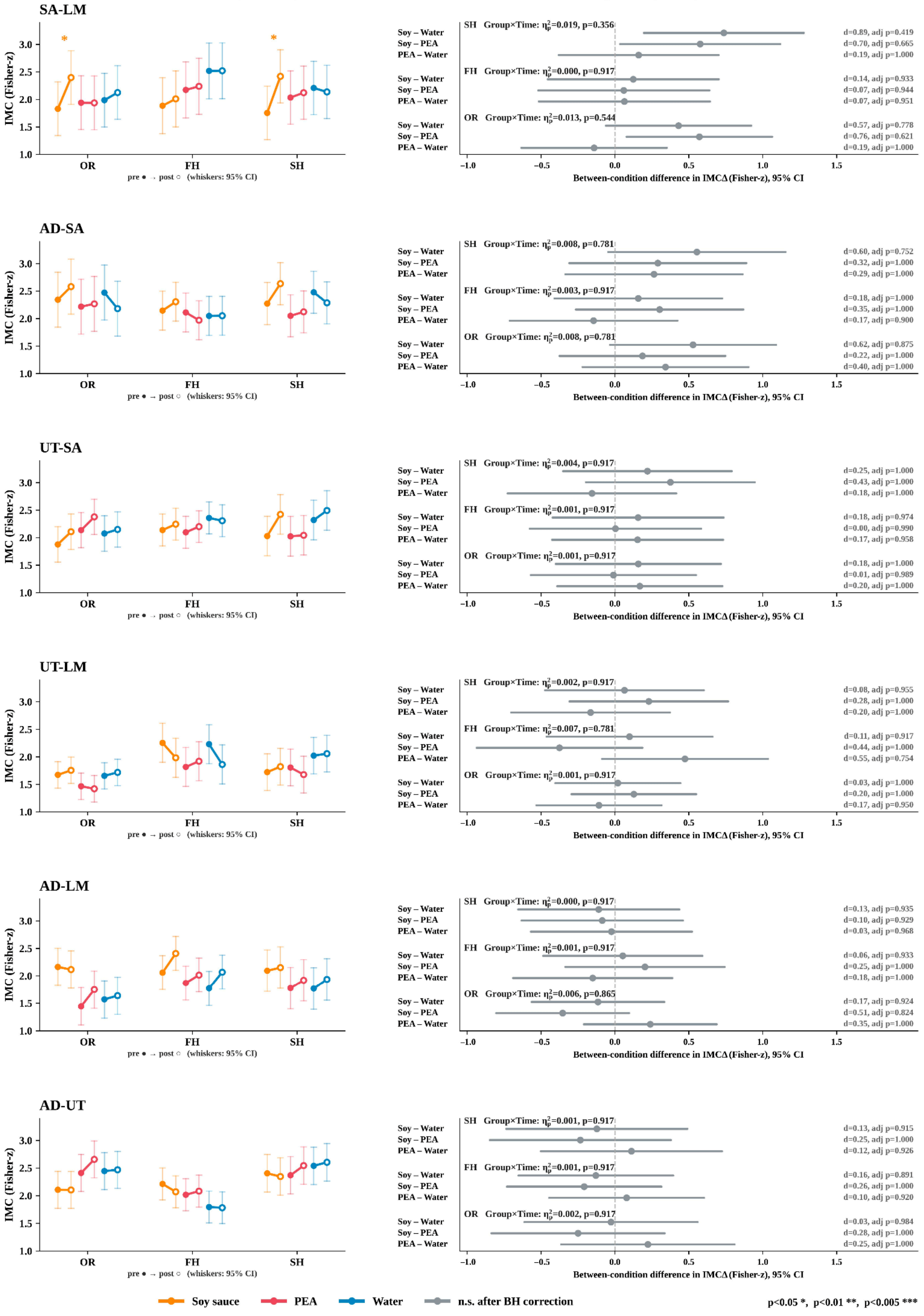

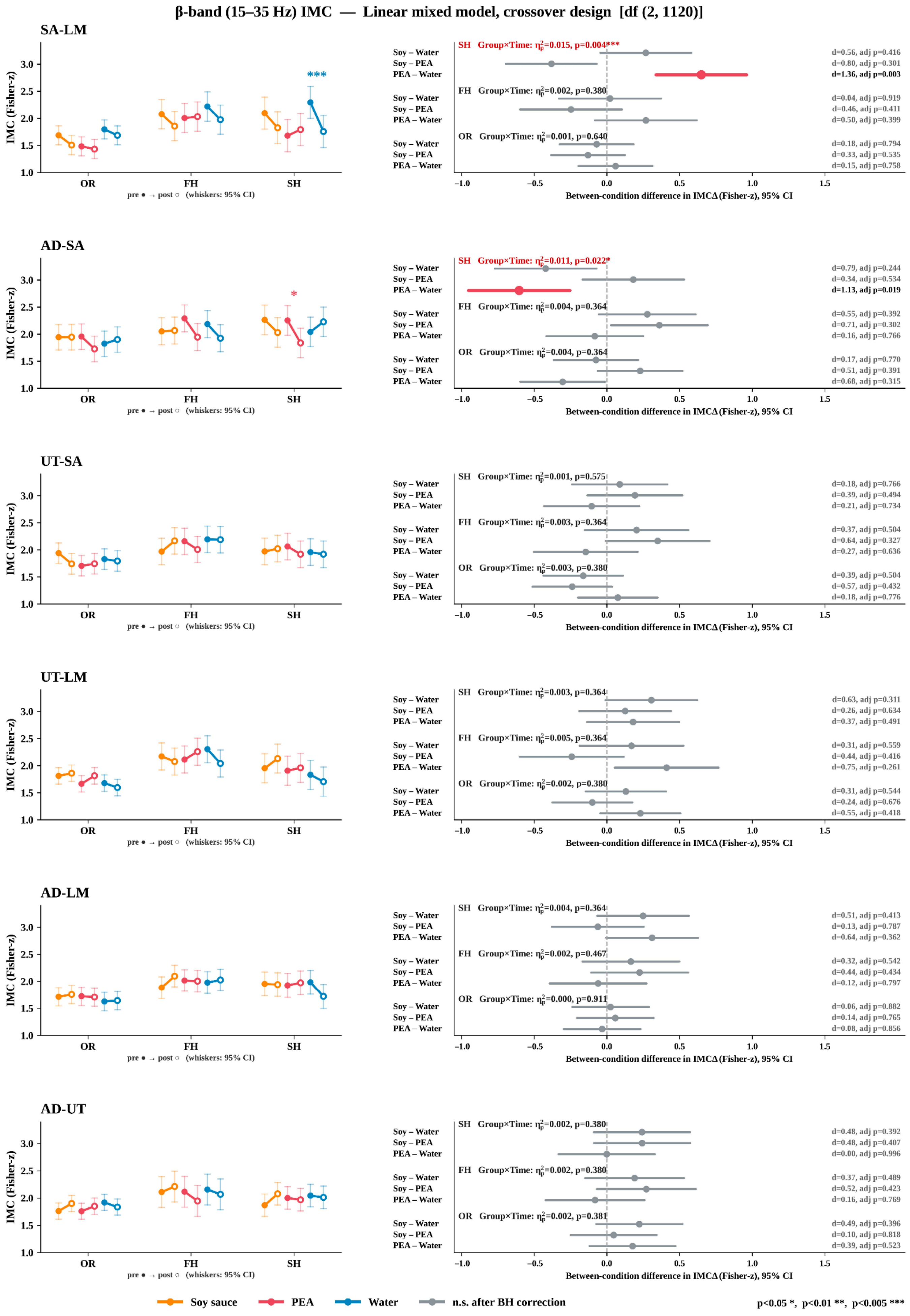
β-band (15–35 Hz) IMC — Linear mixed model, crossover design [df (2, 1120)]
SA-LM
AD-SA
UT-SA
UT-LM
AD-LM
AD-UT
IMC (Fisher-z)
OR
FH
SH
pre ● → post ○ (whiskers: 95% CI)
SA-LM: SH Group×Time: η_p^2=0.015, p=0.004***
Soy – Water d=0.56, adj p=0.416
Soy – PEA d=0.80, adj p=0.301
PEA – Water d=1.36, adj p=0.003
SA-LM: FH Group×Time: η_p^2=0.002, p=0.380
Soy – Water d=0.04, adj p=0.919
Soy – PEA d=0.46, adj p=0.411
PEA – Water d=0.50, adj p=0.399
SA-LM: OR Group×Time: η_p^2=0.001, p=0.640
Soy – Water d=0.18, adj p=0.794
Soy – PEA d=0.33, adj p=0.535
PEA – Water d=0.15, adj p=0.758
AD-SA: SH Group×Time: η_p^2=0.011, p=0.022*
Soy – Water d=0.79, adj p=0.244
Soy – PEA d=0.34, adj p=0.534
PEA – Water d=1.13, adj p=0.019
AD-SA: FH Group×Time: η_p^2=0.004, p=0.364
Soy – Water d=0.55, adj p=0.392
Soy – PEA d=0.71, adj p=0.302
PEA – Water d=0.16, adj p=0.766
AD-SA: OR Group×Time: η_p^2=0.004, p=0.364
Soy – Water d=0.17, adj p=0.770
Soy – PEA d=0.51, adj p=0.391
PEA – Water d=0.68, adj p=0.315
UT-SA: SH Group×Time: η_p^2=0.001, p=0.575
Soy – Water d=0.18, adj p=0.766
Soy – PEA d=0.39, adj p=0.494
PEA – Water d=0.21, adj p=0.734
UT-SA: FH Group×Time: η_p^2=0.003, p=0.364
Soy – Water d=0.37, adj p=0.504
Soy – PEA d=0.64, adj p=0.327
PEA – Water d=0.27, adj p=0.636
UT-SA: OR Group×Time: η_p^2=0.003, p=0.380
Soy – Water d=0.39, adj p=0.504
Soy – PEA d=0.57, adj p=0.432
PEA – Water d=0.18, adj p=0.776
UT-LM: SH Group×Time: η_p^2=0.003, p=0.364
Soy – Water d=0.63, adj p=0.311
Soy – PEA d=0.26, adj p=0.634
PEA – Water d=0.37, adj p=0.491
UT-LM: FH Group×Time: η_p^2=0.005, p=0.364
Soy – Water d=0.31, adj p=0.559
Soy – PEA d=0.44, adj p=0.416
PEA – Water d=0.75, adj p=0.261
UT-LM: OR Group×Time: η_p^2=0.002, p=0.380
Soy – Water d=0.31, adj p=0.544
Soy – PEA d=0.24, adj p=0.676
PEA – Water d=0.55, adj p=0.418
AD-LM: SH Group×Time: η_p^2=0.004, p=0.364
Soy – Water d=0.51, adj p=0.413
Soy – PEA d=0.13, adj p=0.787
PEA – Water d=0.64, adj p=0.362
AD-LM: FH Group×Time: η_p^2=0.002, p=0.467
Soy – Water d=0.32, adj p=0.542
Soy – PEA d=0.44, adj p=0.434
PEA – Water d=0.12, adj p=0.797
AD-LM: OR Group×Time: η_p^2=0.000, p=0.911
Soy – Water d=0.06, adj p=0.882
Soy – PEA d=0.14, adj p=0.765
PEA – Water d=0.08, adj p=0.856
AD-UT: SH Group×Time: η_p^2=0.002, p=0.380
Soy – Water d=0.48, adj p=0.392
Soy – PEA d=0.48, adj p=0.407
PEA – Water d=0.00, adj p=0.996
AD-UT: FH Group×Time: η_p^2=0.002, p=0.380
Soy – Water d=0.37, adj p=0.489
Soy – PEA d=0.52, adj p=0.423
PEA – Water d=0.16, adj p=0.769
AD-UT: OR Group×Time: η_p^2=0.002, p=0.381
Soy – Water d=0.49, adj p=0.396
Soy – PEA d=0.10, adj p=0.818
PEA – Water d=0.39, adj p=0.523
Between-condition difference in IMCΔ (Fisher-z), 95% CI
Soy sauce
PEA
Water
n.s. after BH correction
p<0.05 *, p<0.01 **, p<0.005 ***
