# Supplementary material for: Soy Sauce Odor Improves Upper Limb Motor Performance with Preliminary Evidence of Increased Alpha-Band Intermuscular Coherence Between Postural Muscles: An Exploratory Within-Subjects Crossover Study
Source: Brain Sci. 2026 Jul 12;16(7):737. doi: 10.3390/brainsci16070737 (PMC13407258; doi:10.3390/brainsci16070737)
Supplement: Supplementary file 1 [file brainsci-16-00737-s001.zip › brainsci-4265143-Table S1. CONSORT Crossover Checklist.pdf]

Supplementary Materials

Table S1: CONSORT 2010 Checklist — Extension to Crossover Randomised Trials

Trial title: *Soy sauce odor improves upper limb motor performance with preliminary evidence of increased alpha-band intermuscular coherence between postural muscles: An exploratory within-subjects crossover study*

Reference: *Dwan K et al. CONSORT 2010 statement: extension to randomised crossover trials. BMJ* 2019;366:l4378. † Items marked with † are crossover extension items added to the standard CONSORT 2010 checklist.

Section / Topic	Item No.	Checklist Item	Reported on Section / Figure
**TITLE AND ABSTRACT**			
Title and abstract	1a	Identification as a randomised trial in the title; for crossover trials, state that the trial is a crossover design	Title page
	1b	Structured summary of trial design, methods, results, and conclusions (including sample size and exploratory nature)	Abstract
**INTRODUCTION**			
Background and objectives	2a	Scientific background and explanation of rationale	Introduction
	2b	Specific objectives or hypotheses	Introduction (final paragraph)
**METHODS**			
Trial design	3a	Description of trial design (type: crossover) including allocation ratio	Section 2.1
	3b	Important changes to methods after trial commencement, with reasons	Not applicable
	3c†	Number of periods (3) and sequences (6); rationale for use of a crossover design	Section 2.1
Participants	4a	Eligibility criteria for participants (inclusion and exclusion criteria)	Section 2.2
	4b	Settings and locations where the data were collected	Section 2.3.1
Interventions	5	The interventions for each condition with sufficient detail for replication, including how and when administered (1 mL on odor paper; 20 s exposure; direct subnasal presentation)	Section 2.3.1
	5c†	Washout period between intervention periods and justification for its adequacy (≥1 day; range 1–83 days; mean 19.6 days; median 3 days; justified by rapid resolution of olfactory adaptation)	Section 2.1
Outcomes	6a	Completely defined pre-specified primary (mFRT) and secondary/exploratory (IMC: α- and β-bands, 6 muscle pairs, 3 time periods) outcome measures, including how and when assessed	Sections 2.3.3, 2.4
	6b	Any changes to trial outcomes after the trial commenced, with reasons	Not applicable

Section / Topic	Item No.	Checklist Item	Reported on Section / Figure
Sample size	7a	How sample size was determined: G*Power 3.1.9.7; effect size f = 0.40 (based on r = 0.778 from prior study); α = 0.05; power = 0.80; conservative repeated-measures correlation = 0.70 → n = 9. IMC analyses are exploratory (no formal power analysis for IMC endpoints)	Section 2.1; Table S2
	7b	Explanation of any interim analyses and stopping guidelines	Not applicable
Sequence generation	8a	Method used to generate the random allocation sequence	Section 2.1
	8b	Type of randomisation: balanced complete crossover; all 6 possible sequences of 3 odor conditions enumerated a priori; 2 participants assigned per sequence	Section 2.1
Allocation concealment	9	Mechanism used to implement the random allocation sequence; steps to conceal until interventions were assigned (participants blinded to odor identity throughout)	Section 2.1
Implementation	10	Who generated the allocation sequence (PI: Y.Y.), who enrolled participants, and who assigned participants to interventions	Section 2.1
Blinding	11a	Who was blinded after assignment to interventions: participants blinded to odor identity; experimenter administering odor was aware of the assigned condition (open-label for experimenter)	Section 2.1
	11b	Description of the similarity of interventions: all odors presented in identical manner (odor paper, 1 mL solution, 20 s; participants uninformed of content)	Section 2.3.1
	11c†	Whether participants were blinded to their sequence of treatment periods: participants were not informed of the odor presentation order across sessions	Section 2.1
Statistical methods	12a	Two-way repeated-measures ANOVA with post-hoc Bonferroni test for mFRT; Friedman test and Wilcoxon signed-rank test with Bonferroni correction for subjective odor evaluation; LMM for IMC (group × time interaction)	Section 2.5
	12b	Methods for additional analyses: IMC analyzed across three time periods (OR, FH, SH); Fisher z-transformation applied for normalization; subject treated as random factor in LMM; Benjamini–Hochberg (BH) correction applied within each frequency band for multiple post-hoc comparisons	Section 2.5.2
	12c†	Pre-specified method to test for or adjust for carry-over effects	Not pre-specified; carry-over assessed post-hoc by comparing pre-

Section / Topic	Item No.	Checklist Item	Reported on Section / Figure
			intervention mFRT scores across the three periods (Friedman test; Section 3.2); acknowledged as a limitation (Section 5.2)
**RESULTS**			
Participant flow	13a	For each group: number randomly assigned, receiving intended treatment, and analysed for the primary outcome	Figure 2; Section 2.2 (enrolled: n = 12; analysed: n = 9)
	13b	Losses and exclusions after randomisation with reasons (n = 3: 2 EMG data loss; 1 scheduling conflict)	Figure 2; Section 2.2
	13c†	Number of participants included in each period and sequence (all 9 completed all 3 periods)	Figures 1–2
Recruitment	14a	Dates defining the periods of recruitment and follow-up	Ethics approval: 14 June 2018; specific recruitment dates not stated
	14b	Why the trial ended or was stopped early, if applicable	Not applicable
Baseline data	15	Baseline demographic and clinical characteristics for each group (included vs. excluded participants)	Table 1
	15c†	Baseline characteristics for each period, if applicable: pre-odor mFRT equivalence across three odor conditions verified by one-way repeated-measures ANOVA	Section 2.5.1
Numbers analysed	16	Number of participants analysed (n = 9) and whether by originally assigned conditions (complete crossover; all participants contributed data to all conditions)	Section 2.2; Table 1
Outcomes and estimation	17a	For each primary and secondary outcome: results for each group and estimated effect size with precision (95% CI or LSM ± 95% CI)	Table 3 (mFRT pre/post with effect estimates); Figures 8–9 and Figure S1 (IMC, Fisher-z with 95% CI and Cohen's d)
	17b	For binary outcomes: absolute and relative effect sizes	Not applicable (continuous outcomes)
	17c†	Results for each period and estimates of carry-over effects and their precision	Pre-intervention mFRT scores reported by period and carry-over assessed via Friedman test across

Section / Topic	Item No.	Checklist Item	Reported on Section / Figure
			the three periods (Table 2; Section 3.2); period-by-period results for IMC outcomes not separately reported
Ancillary analyses	18	Results of any other analyses performed, including subgroup analyses, distinguishing pre-specified from exploratory: IMC results reported for OR, FH, and SH sub-periods (exploratory)	Section 3.3
Harms	19	All important harms or unintended effects in each group	No adverse events occurred; not formally reported
**DISCUSSION**			
Limitations	20	Trial limitations addressing potential bias, imprecision, and if relevant, multiplicity of analyses (small n; multiplicity addressed by Benjamini–Hochberg correction; exploratory IMC analyses not formally powered; carry-over assessed but not pre-specified; open-label experimenter; short/variable washout; male-only cohort)	Section 5.2
Generalisability	21	Generalisability (external validity) of the trial findings (healthy young Japanese males; single cultural/dietary context)	Section 5.2
Interpretation	22	Interpretation consistent with results, balancing benefits and harms, and considering other relevant evidence	Sections 4, 5.1
**OTHER INFORMATION**			
Registration	23	Registration number and name of trial registry	Not registered
Protocol	24	Where the full trial protocol can be accessed, if available	The full trial protocol will be made available through the institutional repository of University of Kochi Health Sciences upon publication.
Funding	25	Sources of funding and other support; role of funders	Funding section (JSPS KAKENHI Grant No. 25K24359)
