# Supplementary material for: Soy Sauce Odor Improves Upper Limb Motor Performance with Preliminary Evidence of Increased Alpha-Band Intermuscular Coherence Between Postural Muscles: An Exploratory Within-Subjects Crossover Study
Source: Brain Sci. 2026 Jul 12;16(7):737. doi: 10.3390/brainsci16070737 (PMC13407258; doi:10.3390/brainsci16070737)
Supplement: Supplementary file 1 [file brainsci-16-00737-s001.zip › brainsci-4265143-Table S2. Effect Size Calculation from Previous Study.pdf]

Supplementary Material

Table S2: Effect Size Calculation from Previous Study.

Parameter	Value
P-value	0.0001363
Effect Size (*r*)	0.7787 (Large)
Z	3.8149
W, (W-, W+)	16, (16, 284)
Number of pairs (*N*)	24
Non-zero difference pairs (*n*)	24
Ties Correction	0.375
S.E	34.9946
Average of differences ($\bar{x}_d$)	35.0417
SD of differences (S_d)	30.4395
Normality p-value	0.9874
Skewness	0.02612

Results of the Wilcoxon Signed-Rank test indicated that there is a significant large difference between Pre (*Mdn* = 433.5, *n* = 24) and Post (*Mdn* = 456, *n* = 24), $Z = 3.8$, $p < .001$, $r = 0.8$.

Calculated based on prior research. Detailed parameters and validation are available in our previous report (Yano et al., 2019).

https://onlinelibrary.wiley.com/doi/10.1155/2019/2748721

The Wilcoxon Signed-Rank calculator calculates the Wilcoxon Signed-Rank W

**Pre – mTRT (mm)**	**Post – mFRT (mm)**	**Difference**	**Abs Diff**	**Sign**	**Rank – Abs diff**	**W-**	**W+**
435	457	22	22	+	7		**7**
285	335	50	50	+	17		**17**
359	412	53	53	+	19		**19**
287	359	72	72	+	21		**21**
299	313	14	14	+	5		**5**
452	484	32	32	+	12		**12**
383	411	28	28	+	10		**10**
470	459	-11	11	-	4	**4**	
360	458	98	98	+	24		**24**
372	451	79	79	+	23		**23**
441	412	-29	29	-	11	**11**	
454	480	26	26	+	8.5		**8.5**
505	539	34	34	+	13		**13**
429	455	26	26	+	8.5		**8.5**
487	539	52	52	+	18		**18**
522	593	71	71	+	20		**20**
356	377	21	21	+	6		**6**
436	477	41	41	+	15.5		**15.5**
426	464	38	38	+	14		**14**
379	452	73	73	+	22		**22**
439	447	8	8	+	2.5		**2.5**
432	426	-6	6	-	1	**1**	
529	537	8	8	+	2.5		**2.5**
447	488	41	41	+	15.5		**15.5**
**Total:**						16	284
