# Supplementary material for: Soy Sauce Odor Improves Upper Limb Motor Performance with Preliminary Evidence of Increased Alpha-Band Intermuscular Coherence Between Postural Muscles: An Exploratory Within-Subjects Crossover Study
Source: Brain Sci. 2026 Jul 12;16(7):737. doi: 10.3390/brainsci16070737 (PMC13407258; doi:10.3390/brainsci16070737)
Supplement: Supplementary file 1 [file brainsci-16-00737-s001.zip › brainsci-4265143-Table S3. Subjective evaluation of odors comparison among three odors.pdf]

Supplementary Materials

Table S3. Subjective evaluation of odors: comparison among three odors.

Measure	χ^2 (Degrees of Freedom)	p -value
Appetizing	17.03 (2)	$< 0.001^{****}$
Familiarity	15.79 (2)	$< 0.001^{****}$
Pleasantness	13.61 (2)	$< 0.001^{****}$
Relaxation	14.25 (2)	$< 0.001^{****}$
Intensity	14.35 (2)	$< 0.001^{****}$

****, $p < 0.001$; Friedman test.
