# Supplementary material for: Soy Sauce Odor Improves Upper Limb Motor Performance with Preliminary Evidence of Increased Alpha-Band Intermuscular Coherence Between Postural Muscles: An Exploratory Within-Subjects Crossover Study
Source: Brain Sci. 2026 Jul 12;16(7):737. doi: 10.3390/brainsci16070737 (PMC13407258; doi:10.3390/brainsci16070737)
Supplement: Supplementary file 1 [file brainsci-16-00737-s001.zip › brainsci-4265143-Table S4. Subjective evaluation of odors comparison between two odors.pdf]

Supplementary Materials

Table S4. Subjective evaluation of odors: comparison between two odors.

Measure	Comparison	*p* -value (corrected)
Appetizing	Soy Sauce vs. PEA	0.010**
	Soy Sauce vs. Water	0.002***
	PEA vs. Water	1.00
Familiarity	Soy Sauce vs. PEA	0.029*
	Soy Sauce vs. Water	0.002***
	PEA vs. Water	1.00
Pleasantness	Soy Sauce vs. PEA	0.178
	Soy Sauce vs. Water	0.377
	PEA vs. Water	0.002***
Relaxation	Soy Sauce vs. PEA	0.102
	Soy Sauce vs. Water	0.472
	PEA vs. Water	0.002***
Intensity	Soy Sauce vs. PEA	1.00
	Soy Sauce vs. Water	0.003***
	PEA vs. Water	0.007***

*, $p < 0.05$; **, $p < 0.01$; ***, $p < 0.005$. Wilcoxon signed-rank test with Bonferroni correction.
