# Supplementary material for: Soy Sauce Odor Improves Upper Limb Motor Performance with Preliminary Evidence of Increased Alpha-Band Intermuscular Coherence Between Postural Muscles: An Exploratory Within-Subjects Crossover Study
Source: Brain Sci. 2026 Jul 12;16(7):737. doi: 10.3390/brainsci16070737 (PMC13407258; doi:10.3390/brainsci16070737)
Supplement: Supplementary file 1 [file brainsci-16-00737-s001.zip › brainsci-4265143-Table S5. Cohens-d/partial eta2.pdf]

Supplementary Materials

Table S5. Cohen's *d* for within-condition pre–post comparisons, partial η² for Group × Time interactions, and corresponding Benjamini-Hochberg (BH)-adjusted *p* values for intermuscular coherence (IMC).

P values and effect sizes are derived from linear mixed model analyses with odor condition (Group: soy sauce, PEA, water) and time (pre- vs. post-odor) as fixed factors and subject as a random factor. For within-condition pre–post comparisons, Cohen's *d* is shown, and BH-adjusted *p* values for these comparisons are also shown. For Group × Time interactions, partial η² is shown, together with the corresponding BH-adjusted *p* values. BH-adjusted *p* values were computed using the BH procedure (54 comparisons per frequency band). Bold values indicate adjusted $p < 0.05$. AD, deltoid; SA, serratus anterior; UT, upper trapezius; LM, lumbar multifidus; OR, overall reach; FH, first half of reach; SH, second half of reach; PEA, phenylethyl alcohol.

α-band (8–14 Hz)

		Within-condition pre–post comparison						Group × Time interaction	
		Water		Soy sauce		PEA			
**Period**	**Muscle pair**	**Cohen's *d***	***Adjusted p***	**Cohen's *d***	***Adjusted p***	**Cohen's *d***	***Adjusted p***	**partial η²**	***Adjusted p***
Overall reach (OR)	SA×LM	0.259	0.951	1.068	**0.038**	0.005	0.995	0.013	0.544
	AD×SA	0.480	0.898	0.390	0.951	0.084	0.951	0.008	0.781
	UT×SA	0.120	0.951	0.382	0.951	0.399	0.951	0.001	0.917
	UT×LM	0.136	0.951	0.180	0.951	0.100	0.951	0.001	0.917
	AD×LM	0.138	0.951	0.098	0.951	0.629	0.648	0.006	0.865
	AD×UT	0.037	0.995	0.005	0.995	0.388	0.951	0.002	0.917
First half (FH)	SA×LM	0.002	0.995	0.200	0.951	0.104	0.951	< 0.001	0.917
	AD×SA	0.005	0.995	0.262	0.951	0.231	0.951	0.003	0.917
	UT×SA	0.080	0.951	0.170	0.951	0.164	0.951	0.001	0.917
	UT×LM	0.609	0.648	0.447	0.951	0.171	0.951	0.007	0.781
	AD×LM	0.509	0.855	0.599	0.648	0.251	0.951	0.001	0.917
	AD×UT	0.023	0.995	0.252	0.951	0.116	0.951	0.001	0.917
Second half (SH)	SA×LM	0.123	0.951	1.135	**0.038**	0.151	0.951	0.019	0.356
	AD×SA	0.296	0.951	0.559	0.719	0.112	0.951	0.008	0.781
	UT×SA	0.282	0.951	0.638	0.648	0.030	0.995	0.004	0.917
	UT×LM	0.062	0.979	0.173	0.951	0.221	0.951	0.002	0.917
	AD×LM	0.283	0.951	0.097	0.951	0.243	0.951	< 0.001	0.917
	AD×UT	0.096	0.951	0.088	0.951	0.265	0.951	0.001	0.917

β-band (15–35 Hz)

		Within-condition pre–post comparison						Group × Time interaction	
		Water		Soy sauce		PEA			
**Period**	**Muscle pair**	**Cohen's *d***	***Adjusted p***	**Cohen's *d***	***Adjusted p***	**Cohen's *d***	***Adjusted p***	**partial η²**	***Adjusted p***
Overall reach (OR)	SA×LM	0.402	0.535	0.658	0.263	0.185	0.882	0.001	0.640
	AD×SA	0.243	0.744	0.004	0.989	0.721	0.234	0.004	0.364
	UT×SA	0.119	0.904	0.669	0.263	0.135	0.882	0.003	0.380
	UT×LM	0.273	0.744	0.165	0.882	0.501	0.393	0.002	0.380
	AD×LM	0.060	0.958	0.149	0.882	0.054	0.958	< 0.001	0.911
	AD×UT	0.264	0.744	0.433	0.499	0.287	0.744	0.002	0.381

First half (FH)	SA×LM	0.641	0.268	0.583	0.289	0.067	0.958	0.002	0.380
	AD×SA	0.727	0.234	0.044	0.958	0.958	0.074	0.004	0.364
	UT×SA	0.016	0.979	0.513	0.393	0.393	0.536	0.003	0.364
	UT×LM	0.682	0.263	0.244	0.744	0.381	0.547	0.005	0.364
	AD×LM	0.135	0.882	0.593	0.289	0.032	0.958	0.002	0.467
	AD×UT	0.241	0.744	0.276	0.744	0.461	0.449	0.002	0.380
Second half (SH)	SA×LM	1.591	**< 0.001**	0.798	0.226	0.331	0.642	0.015	**0.004**
	AD×SA	0.494	0.393	0.622	0.280	1.103	**0.026**	0.011	**0.022**
	UT×SA	0.108	0.915	0.142	0.882	0.403	0.535	0.001	0.575
	UT×LM	0.372	0.550	0.523	0.392	0.155	0.882	0.003	0.364
	AD×LM	0.768	0.926	0.039	0.958	0.142	0.882	0.004	0.364
	AD×UT	0.089	0.926	0.585	0.289	0.092	0.926	0.002	0.380
